# Inhibition of Pyroptosis by Hydroxychloroquine as a Neuroprotective Strategy in Ischemic Stroke

**DOI:** 10.1523/ENEURO.0254-24.2024

**Published:** 2025-01-03

**Authors:** Wenshuo Peng, Kaiming Guo, Jian Hu, Qianchun Wang

**Affiliations:** ^1^Department of Pharmacy, The First Afﬁliated Hospital of Wenzhou Medical University, Wenzhou 325015, China; ^2^School of Pharmaceutical Sciences, Wenzhou Medical University, Wenzhou 325015, China; ^3^Department of pharmacy, The Second Affiliated Hospital of Nanjing Medical University, Nanjing 210000, China; ^4^Department of gastroenterology, The First affiliated hospital of Wenzhou Medical University, Wenzhou 325015, China

**Keywords:** cell pyroptosis, hydroxychloroquine, ischemic stroke, microglial cells, neuroprotection

## Abstract

Hydroxychloroquine (HCQ), a well-known antimalarial and anti-inflammatory drug, has demonstrated potential neuroprotective effects in ischemic stroke by inhibiting pyroptosis, a programmed cell death associated with inflammation. This study investigates the impact of HCQ on ischemic stroke pathology using both in vivo and in vitro models. In vivo, C57BL/6 mice subjected to middle cerebral artery occlusion (MCAO) were treated with HCQ. Neurological deficits, infarct volume, and the expression of pyroptosis markers were evaluated. The results demonstrated that HCQ significantly improved motor function and reduced infarct volume in the MCAO mouse model. In vitro, BV2 microglial cells exposed to lipopolysaccharide (LPS) and oxygen–glucose deprivation (OGD) were treated with HCQ. Western blot and immunofluorescence analyses revealed that HCQ effectively suppressed the expression of pyroptosis markers GSDMD and NLRP3 in both in vivo and in vitro models. These findings suggest that HCQ mitigates ischemic stroke damage by inhibiting pyroptosis, highlighting its potential as a therapeutic agent for ischemic stroke. This study provides novel insights into the molecular mechanisms by which HCQ exerts its neuroprotective effects, offering a promising new avenue for developing safe, cost-effective, and widely applicable stroke treatments. The potential of HCQ to modulate neuroinflammatory pathways presents a significant advancement in ischemic stroke therapy, emphasizing the importance of targeting pyroptosis in stroke management and the broader implications for treating neuroinflammatory conditions.

## Significance Statement

Ischemic stroke remains a leading cause of disability and death globally, with limited effective treatments. This study reveals that HCQ significantly mitigates ischemic stroke damage by inhibiting pyroptosis, a form of programmed cell death. Using in vivo and in vitro models, HCQ was shown to improve motor function and reduce infarct volume, highlighting its potential as a neuroprotective agent. These findings offer a promising new therapeutic approach for ischemic stroke, emphasizing the importance of targeting pyroptosis in stroke treatment.

## Introduction

Stroke poses a significant challenge in the global health domain. According to the World Health Organization, millions of deaths occur annually due to stroke, ranking it among the top 3 causes of global mortality (Biancari et al., 2024a,b). Ischemic stroke, constituting ∼80% of all stroke cases, results from inadequate blood supply to the brain, leading to functional impairment or cell death (Wei et al., 2021; Zhu et al., 2022). This type of stroke is mainly attributed to vascular blockages such as thrombus formation or arterial sclerosis, causing interruptions in blood flow (Hayakawa et al., 2023). The high incidence, disability rate, and recurrence rate of ischemic stroke have long been focal points in global public health (GBD 2019 Stroke Collaborators, 2021; Ding et al., 2022; Tu et al., 2023). Apart from imposing a heavy burden on patients and their families, it also exerts significant economic pressure on society and healthcare systems (Strilciuc et al., 2021; Lauder et al., 2023). Thus, a profound understanding of its pathophysiological mechanisms and exploring more effective treatment approaches is crucial for enhancing patient quality of life and alleviating societal burdens (Tater and Pandey, 2021; Qin et al., 2022; Fang et al., 2023).

Although there have been advancements in the treatment of ischemic stroke, existing therapies, such as thrombolysis and anticoagulant treatments, are often constrained by time limitations, patient eligibility criteria, and potential complications (Ardila Jurado et al., 2022; Li et al., 2023). For instance, tissue plasminogen activator (tPA) is the only approved drug for acute ischemic stroke treatment, but its use is restricted to a few hours after onset and is not suitable for all patients (Hasan et al., 2021; Fang et al., 2023; Tsivgoulis et al., 2023). Moreover, the high cost of treatment and the increased need for long-term medical care exacerbate the financial burden on patients (Gao et al., 2022; Benković et al., 2023; Omari et al., 2023). Hence, developing novel therapeutic strategies, particularly those that are safe, cost-effective, and widely applicable, holds paramount significance in improving the prognosis of ischemic stroke patients (Menon et al., 2022; Mosconi and Paciaroni, 2022; Tsivgoulis et al., 2023).

Cell death through pyroptosis is a programmed form that differs from conventional apoptosis by releasing cellular contents, potentially triggering inflammatory reactions in surrounding cells (Meng et al., 2022; Dal and Aru, 2023). In ischemic stroke, pyroptosis plays a pivotal role in the process of neuronal damage and neuroinflammation (Vorobeva, 2022). Particularly in the poststroke inflammatory response, microglial cells, the primary immune cells in the brain, exhibit dual functionalities (Greuel et al., 2024). On the one hand, they can clear debris from necrotic and apoptotic cells, while on the other hand, overactivated microglia may release inflammatory mediators, exacerbating brain tissue damage (Merighi et al., 2022; Kitaoka, 2023). Therefore, modulating the activity of microglial cells and inhibiting their excessive responses may present a novel strategy for treating ischemic stroke (Zhu et al., 2021; Candelario-Jalil et al., 2022; Alsbrook et al., 2023).

Hydroxychloroquine (HCQ) is a well-established drug for treating malaria and autoimmune diseases. Studies have indicated that HCQ can penetrate the blood–brain barrier in recent years, making it a potential neuroprotective agent (Ramos et al., 2022; Giuliano et al., 2023). Although research on the neuroprotective effects of HCQ is limited, its application in other systemic diseases suggests that HCQ exerts a wide range of biological actions and may be beneficial in the treatment of ischemic stroke (Berkman and Tapson, 2021; Liu et al., 2023).

This study explores the potential application of HCQ in treating ischemic stroke, specifically focusing on its inhibitory effect on microglial cell pyroptosis. By utilizing a mouse model of middle cerebral artery occlusion (MCAO) and an in vitro model of lipopolysaccharide (LPS)-stimulated microglia, we systematically assessed the impact of HCQ on neurological deficits, infarct volume, and its efficacy in modulating the expression of relevant pyroptosis markers. This research contributes to understanding the mechanisms of action of HCQ in inflammation and neuroprotection and offers new perspectives and strategies for treating ischemic stroke. Given the limited options currently available for treating ischemic stroke, the results of this study may hold significant clinical implications, especially in terms of safety, economy, and ease of administration, providing a scientific basis and innovative approach for future drug development and treatment optimization.

## Materials and Methods

### Ethical statement

This study strictly adheres to internationally recognized animal welfare and ethics guidelines. The experimental protocol was approved by the Ethics Committee of Wenzhou Medical University. All research procedures strictly adhered to the guidelines for the care and use of laboratory animals.

### Identification of HCQ potential target proteins

This study performed searches across three major bioinformatics databases: SwissTargetPrediction (http://www.swisstargetprediction.ch/), DrugBank (https://go.drugbank.com/), and PharmMapper (https://www.lilab-ecust.cn/pharmmapper/). In SwissTargetPrediction, *Homo sapiens* was selected as the species, and HCQ's isomeric SMILES [CCN(CC)CCCC(C)NC1=C2C=CC(=CC2=NC=C1)Cl] was input to predict potential targets based on ligand similarity, selecting the top 100 targets with Tanimoto similarity scores >0.8. In DrugBank, HCQ's CAS number (118-42-3) was entered using the advanced search function to filter known and predicted targets relevant to *Homo sapiens* and retrieve detailed information on targets and their mechanisms of action. In PharmMapper, HCQ's 3D molecular structure was uploaded, restricting targets to human proteins and selecting the top 300 targets with the highest fit scores. The targets identified from these databases were subsequently deduplicated to obtain the most biologically relevant targets.

### Gathering genetic information related to ischemic stroke

This study conducted in-depth searches using two databases, GeneCards (https://www.genecards.org/) and OMIM (https://www.omim.org/), to collect genetic information related to ischemic stroke. By utilizing “ischemic stroke” and “Homo sapiens” as keywords, a total of 4,684 human genes associated with ischemic stroke were identified, laying a solid foundation for further analysis.

### Analysis of key target proteins and enrichment analysis of GO and KEGG pathways

This study utilized a Venn diagram to analyze the common target proteins between HCQ and ischemic stroke. Subsequently, the Gene Ontology (GO) and Kyoto Encyclopedia of Genes and Genomes (KEGG) enrichment analyses were performed using the DAVID database (https://david.ncifcrf.gov/) to identify the molecular functions of the 71 shared target genes and their involvement in biological systems. This step not only aids in a thorough understanding of the potential mechanisms of HCQ in the treatment of ischemic stroke but also provides essential molecular target information for subsequent experimental design and research.

### Experimental animals

For this study, healthy adult male C57BL/6 mice aged 8–10 weeks and weighing 20–25 g were selected. The mice were purchased from Experimental Animal Technology and housed in the SPF-grade Animal Experimental Center at the University. They were kept in cages under a constant temperature of 25°C, with a relative humidity of 50%, and subjected to a 12 h light/dark cycle with *ad libitum* access to food and water. All research procedures strictly adhered to the guidelines for the care and use of laboratory animals. The mice were randomly assigned to each experimental group using a randomization process. Two researchers who were blinded to the treatment conditions performed data analysis.

### Animal grouping for experiment

Before the experiment, all experimental animals were fed a standard diet for 1 week. Healthy adult mice were then randomly divided into three groups: Sham group, MCAO injury group, and HCQ treatment group, each consisting of 8 mice. In the Sham group, no suture occlusion was performed. The MCAO injury group was modeled by middle cerebral artery occlusion (MCAO) surgery in mice. For the treatment group, intraperitoneal injections of 50 mg/kg HCQ were administered every other day, beginning on the first day after modeling, and the therapeutic effects were evaluated on Days 3 and 5. Longer time points were also assessed to determine whether HCQ has a cumulative effect (Zheng et al., 2021). The sham and MCAO injury groups received intraperitoneal injections equal to 0.9% sodium chloride solution.

### Establishment of the MCAO animal model in mice with ischemic stroke

Animals were anesthetized by intramuscular injection of ketamine/xylazine (80/20 mg/kg). Additionally, atropine (0.05 mg/kg) was administered subcutaneously during the surgery. While under anesthesia, the mice were placed supine, and a midline incision was made along the ventral aspect of the neck. Subsequently, the right common carotid artery (CCA), external carotid artery (ECA), and internal carotid artery (ICA) were exposed. The ECA was ligated, and arterial clips were placed on the CCA and ICA to block blood flow. A small incision was made in the ECA, and a guide wire was inserted into the distal end of the ECA and advanced into the middle cerebral artery until resistance was felt. A knot was tied on the ECA to secure the guide wire, then the clip on the CCA was removed. The incision was closed with sutures, and the animals were placed in a warm environment at 37°C for recovery. One hour after MCAO, the mice were re-anesthetized using the same method. The sutures were cut, the ligature on the proximal end of the ECA was removed, the skin was sutured with silk thread, and the wound site was disinfected with iodine. The mice were kept warm and placed back in their cages in a head-down, tail-up position until they regained consciousness at 26°C, and then housed in appropriate conditions. Neurological function assessments were conducted on the mice at 1, 3, 5, 7, 14, and 21 d postsurgery, followed by anesthesia, brain tissue collection, and subsequent experiments.

### Neurobehavioral function evaluation

In this study, a modified version of the neurological severity score (mNSS), rotarod test, grip strength test, and foot fault test were utilized to evaluate the neurological deficits in mice following MCAO at 1, 3, 5, 7, 14, and 21 d poststroke. This comprehensive approach allowed a thorough assessment of the mice's neurobehavioral impairments throughout the poststroke period.

### mNSS score

The mNSS evaluates mouse neurological function from multiple perspectives including limb movement, sensation, reflexes, and balance. This study primarily focuses on assessments related to motor function and balance. The evaluations were conducted using a double-blind method, with scores ranging from 0 to 18, where higher scores indicate greater neurological impairment in mice. Mice that scored 0 on the first-day postsurgery in the MCAO ischemic model were considered failed models and were excluded from subsequent experiments. Specific scoring items and their corresponding values are detailed in Table 1.

**Table 1.** mNSS scoring criteria

Scoring item	Score
Lifting the mouse by the tail	
Forelimb flexion	1
Hindlimb flexion	1
Head turning >10° within 30 s	1
Free walking on the ground	
Normal	0
Difficulty walking in a straight line	1
Circling toward the injured side	2
Leaning toward the injured side	3
Sensory test	
Vision test	1
No contraction activity when pressing the forepaw	1
Beam balance test	
Passing quickly	0
Passing while clinging to the beam	1
One limb hanging down	2
Two limbs hanging down	3
Falls within >40 s	4
Falls within >20 s	5
Falls within 20 s	6
Reflex activity	
No head turn when lightly touching the external ear canal with a cotton swab	1
No blinking when lightly touching the eye with a cotton swab	1
Involuntary torsional spasm	1
No reflex activity when startled	1

### Rotarod experiment

The time that mice spent on the revolving rod was used as an assessment metric to evaluate their motor coordination ability. In the 3 d before the MCAO induction, mice underwent an adaptation training period at a rotation speed of 10 revolutions per minute (rpm). Following the mice's adaptation to the rotating rod, they underwent four progressive acceleration tests, where the speed increased from 10 to 40 rpm over a duration of 5 min. Post-MCAO injury on Days 1, 3, 5, 7, 14, and 21, each mouse was subjected to the same acceleration test thrice daily. After each test session, mice were allowed a half-hour rest before the subsequent evaluation. The mice's average time on the rotating rod was then recorded and analyzed for each group.

### Grip strength test

The muscle strength of mice was measured using a grip strength meter. The mice could grab the grip strength bar with their front paws and gently pull it backward until releasing it. The maximum force applied to the bar while the mice gripped it was recorded. Each mouse underwent three grip strength measurements, and the average was calculated and compared with the grip strength value from the day before the surgery. The grip strength was calculated as follows: Grip Strength (%) = Postoperative average grip strength on day *n* / Preoperative average grip strength × 100%.

### Treadmill experiment

In this experiment, mice were placed on a grid (60 cm × 40 cm, with a grid diameter of 2 × 2 cm and a height of 50 cm above the ground) to walk freely. Healthy mice would grip the grid and walk swiftly and steadily in all directions. In contrast, ischemic mice exhibited slower walking speeds and tended to drop their injured limb into the grid during movement, which was considered a mistake when a front limb fell into the grid. The number of times a mouse fell into the grid while taking 100 steps were recorded to calculate the percentage of falls compared with the total number of steps taken.

### The use of 2,3,5-triphenyltetrazolium chloride staining method

The 2,3,5-triphenyltetrazolium chloride (TTC) staining method is widely used to assess brain ischemia's severity. Mice are rapidly killed under anesthesia, and their brains are promptly collected, frozen at −20°C for 15 min, sliced into 2 mm thick coronal sections, and then stained with a 2% solution of TTC (Sigma-Aldrich) at 37°C for 30 min.

### Cell culture under oxygen–glucose deprivation/reoxygenation model

Mouse BV2 microglial cells purchased from Procell were utilized for the experiment. The cells were cultured in Dulbecco's modified Eagle’s medium (DMEM) supplemented with 10% fetal bovine serum (FBS, Invitrogen) and 1% penicillin-streptomycin (Invitrogen) at 37°C in a 5% CO_2_ humidified incubator. Subsequently, the cells were transferred to glucose- and serum-free DMEM and subjected to incubation in an anaerobic chamber equipped with AnaeroPack-Anaero (Mitsubishi Gas Chemical). Following 2 h of indoor hypoxia, the medium was replaced with DMEM, and the cells were reintroduced to normal culture conditions for reoxygenation for 1 h. The control group of microglial cells was cultured under normal oxygen conditions in an incubator during the corresponding time frame.

### Immunofluorescence staining

The mouse cerebral cortex was subjected to antigen retrieval by immersion in a 90°C citrate buffer solution for 10 min, followed by a 1 h blocking step with 5% bovine serum albumin (BSA). Subsequently, the tissues were incubated overnight at 4°C with primary antibodies including Iba1 (ab178846, Abcam, diluted 1:800), GFAP (ab7260, Abcam, diluted 1:800), NeuN (ab177487, Abcam, diluted 1:800), GSDMD (ab219800, Abcam, diluted 1:800), and NLRP3 (ab270449, Abcam, diluted 1:800). The following day, after washing with PBS, the tissues were incubated for 1 h with secondary antibodies including goat anti-rabbit IgG H&L (Alexa Fluor 488; ab150077, Abcam, diluted 1:1,000) and goat anti-rabbit IgG H&L (Alexa Fluor 594; ab150080, Abcam, diluted 1:1,000). DAPI was used for nuclear staining, and the tissues were then imaged and recorded using fluorescence microscopy.

### Protein immunoblotting

Protein extraction from brain tissue samples was performed using a protein extraction buffer containing 1% protease inhibitor and 1% phosphatase inhibitor. After sonication, the protein concentration in the supernatant was determined using a BCA protein quantification assay kit (Beyotime). Protein separation was carried out on 8–12% SDS-PAGE gels. The samples were transferred onto PVDF membranes and blocked in a 5% milk solution. Subsequently, the membranes were incubated overnight at 4°C with the following primary antibodies: NLRP3 (ab263899, Abcam, dilution 1:1,000), GSDMD-FL (ab219800, Abcam, dilution 1:1,000), N-GSDMD (ab227821, Abcam, dilution 1:1,000), caspase-1 p20 (2225, Cell Signaling Technology, dilution 1:1,000), Pro-IL-1β (31202, Cell Signaling Technology, dilution 1:1,000), mIL-1β (63,124, Cell Signaling Technology, dilution 1:1,000), β-Actin (sc-47778, Santa Cruz, dilution 1:1,000), anti-mouse IgG (H+L; 14,709, Cell Signaling Technology, dilution 1:5,000), and anti-rabbit IgG (H+L; 14,708, Cell Signaling Technology, dilution 1:5,000). After three washes, the membranes were incubated at room temperature for 1.5 h with suitable horseradish peroxidase-conjugated secondary antibodies. Subsequently, protein bands were detected using an enhanced chemiluminescence system and analyzed with ImageJ software.

### Statistical data analysis

Data was analyzed using GraphPad Prism 8.0 software (GraphPad Software). All data are presented as mean ± standard deviation (SD) of three independent experiments. Multiple comparisons (more than two groups) were performed using a one-way analysis of variance (ANOVA), followed by Tukey's post hoc test for evaluation. Differences between the two groups were assessed using an unpaired two-tailed Student's *t* test. Statistical significance among experimental results was defined as a *p* value <0.05.

## Results

### Observation of cell pyroptosis phenomenon in brain tissue of MCAO model mice

**Figure 1.**
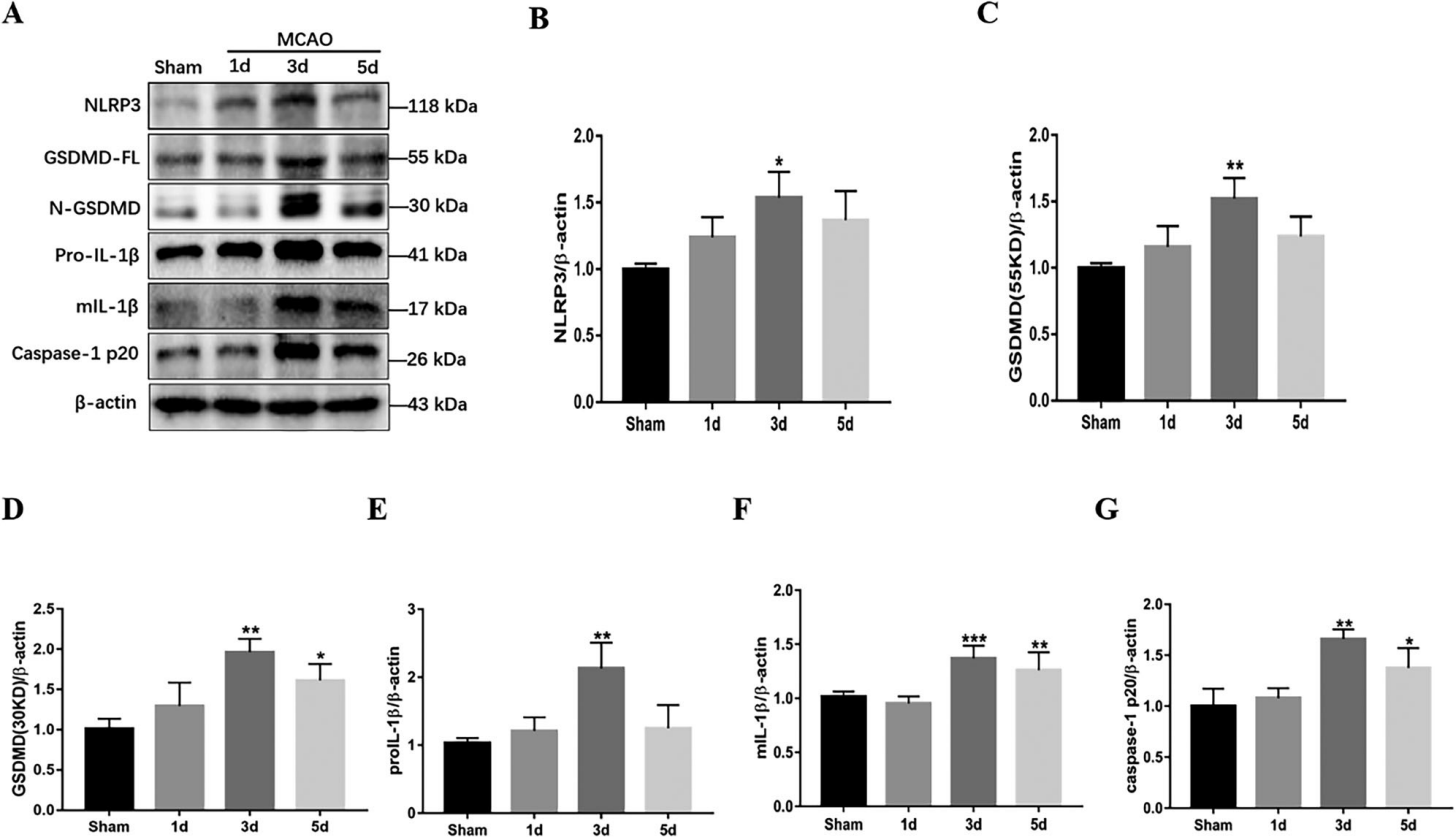
MCAO-induced neuronal cell pyroptosis in mouse brain tissue. ***A***, Protein immunoblot analysis of inflammatory bodies NLRP3, pyroptosis-associated proteins GSDMD-FL, N-GSDMD, Caspase-1 p20, and inflammatory factor IL-1β in mouse brain tissue at 1–5 d post-MCAO treatment. ***B–D***, Western blot quantitative analysis of NLRP3, pyroptosis protein GSDMD-FL, and N-GSDMD. ***E***, ***F***, Quantitative analysis of Pro-IL-1β and mIL-1β by Western blot. ***G***, Quantitative analysis of Caspase-1 by Western blot at Days 1–5 post-MCAO. *n* = 8. Data are presented as mean ± SD. *p < 0.05, **p < 0.01, and ***p < 0.001 compared with the Sham group.

Mice were subjected to MCAO surgery to induce an ischemic stroke model. Brain cortical tissue proteins were extracted from sham-operated (sham group) and MCAO group mice at postoperative days 1, 3, and 5. This study used Western blot technology to examine the proteins NLRP3, GSDMD, IL-1β, and Caspase-1, which are closely associated with inflammasomes and cell pyroptosis (Fig. 1*A*). The results of Western blot analysis revealed a significant increase in the expression levels of inflammasome NLRP3 and pyroptosis-related proteins GSDMD, IL-1β, and Caspase-1 in MCAO group mice compared with the sham-operated group, peaking on postoperative day 3 (Fig. 1*A–G*). Notably, GSDMD, as a crucial initiator of cell pyroptosis, exhibited a dynamic trend of increased expression on Days 1, 3, and 5, reaching its peak on Day 3 (Fig. 1*A*,*C*,*D*). These experimental findings demonstrate that MCAO surgery effectively induces cell pyroptosis in mouse brain tissue, establishing a successful mouse model of ischemic stroke-induced cell pyroptosis.

### GO and pathway enrichment analysis of proteins associated with HCQ and ischemic stroke

**Figure 2.**
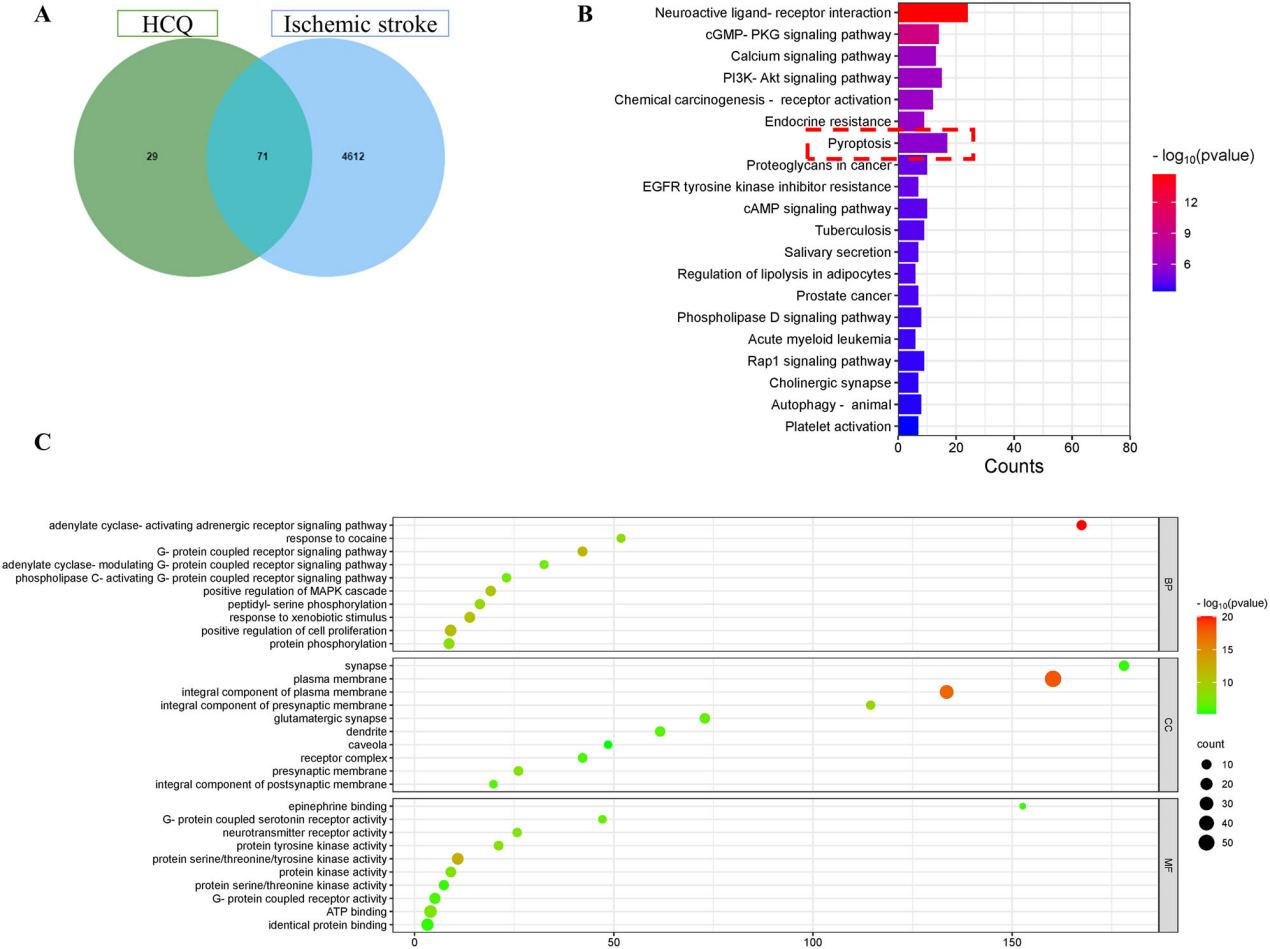
GO and pathway enrichment analysis of proteins associated with HCQ and ischemic stroke. ***A***, A Venn diagram depicting the relationship between predicted target proteins of HCQ and proteins associated with ischemic stroke. ***B***, Results of KEGG analysis for the 71 common genes. ***C***, Results of GO analysis for the 71 common genes.

We intersected the top 100 predicted HCQ target proteins with 4,683 proteins associated with ischemic stroke, resulting in 71 overlapping genes (Fig. 2*A*). These 71 genes underwent GO analysis (Fig. 2*C*) and KEGG pathway enrichment analysis (Fig. 2*B*). A macroscopic biological assessment of these proteins was conducted. According to the GO enrichment analysis, HCQ exerts its biological effects in the network mainly through the adenylyl cyclase-activating adrenaline receptor signaling pathway, response to cocaine, and G-protein-coupled receptor signaling pathway. These proteins are predominantly located at synapses, ion membranes, and ion membrane components. In terms of molecular function, these proteins are primarily involved in adrenaline binding, G-protein-coupled serotonin receptor activity, neurotransmitter receptor activity, and other functions. The KEGG pathway analysis further reveals that these proteins are mainly associated with neuroactive ligand–receptor interactions, cGMP-PKG signaling pathway, calcium signaling pathway, PI3K-Akt signaling pathway, cancer-receptor activation pathway, endocrine resistance, and cell pyroptosis. Subsequent research will focus on the regulatory role of HCQ in cell pyroptosis.

### Improvement of motor function impairment and reduction of brain infarct volume in mice with MCAO by HCQ treatment

**Figure 3.**
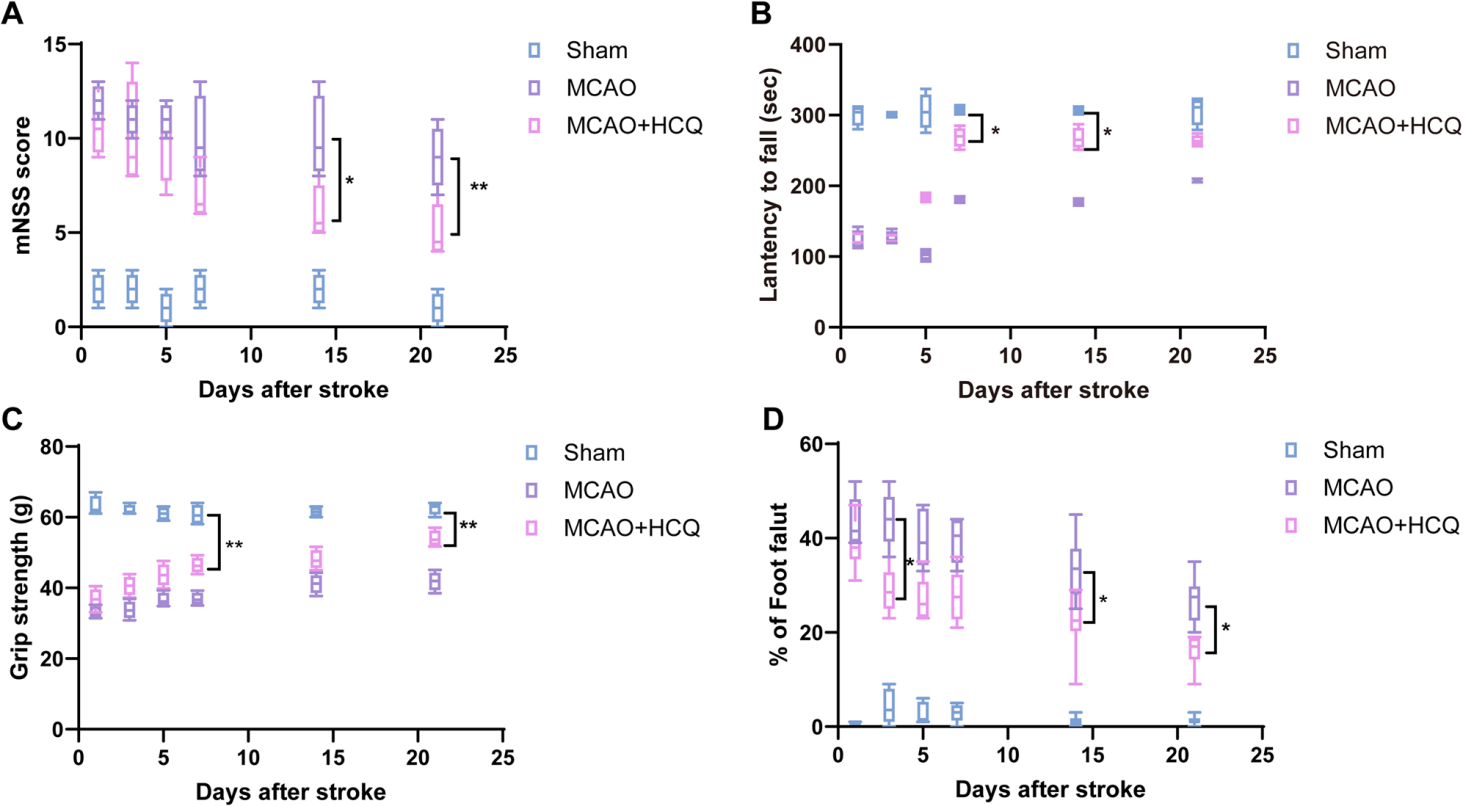
Effect of HCQ on motor and balance function impairment in MCAO mice within 21 d poststroke. ***A***, Impact of HCQ on the mNSS neurofunctional scoring of MCAO mice at 1, 3, 5, 7, 14, and 21 d poststroke. ***B***, Influence of HCQ on the rotarod retention time of MCAO mice at 1, 3, 5, 7, 14, and 21 d poststroke. ***C***, ***D***, Effects of HCQ on the motor balance function of mice poststroke in terms of grip strength (***C***) and foot fault test (***D***) at 1, 3, 5, 7, 14, and 21 d. *p < 0.05, **p < 0.01, compared with the injury group. *n* = 8, data presented as mean ± SD.

This study conducted a series of behavioral tests to assess the potential of HCQ in ameliorating neurological damage and promoting neurofunctional recovery in mice following MCAO, including the rotarod test, mNSS, foot fault test, and grip strength test. In the mNSS assessment, as shown in Figure 3*A*, mice in the MCAO model group exhibited significantly higher neurological function scores from Day 1 post-MCAO than the sham surgery group. Similarly, in the rotarod test, starting from Day 1 post-MCAO, the model group mice displayed significantly shorter latency times on the rotarod compared with the sham group, with the difference becoming more pronounced over time. By Day 21, the difference between the two groups was most significant. Conversely, in comparison with the MCAO model group, HCQ-treated MCAO mice showed a gradual increase in retention time on the rotarod starting from Day 5, with a significant improvement observed on Day 7 (Fig. 3*B*). These results indicate that MCAO injury leads to substantial impairments in motor and balance functions, while HCQ treatment significantly improves these deficits, suggesting a neuroprotective role of HCQ against MCAO-induced neuronal damage.

Moreover, observations on Days 3, 5, and beyond suggest that the neuroprotective effect of HCQ has a cumulative effect. Additionally, muscle strength was evaluated using a grip strength test to assess HCQ's ability to mitigate MCAO-induced muscle weakness. The results showed that MCAO caused significant muscle atrophy and a decline in grip strength, but HCQ treatment markedly improved the muscle atrophy and increased grip strength, progressively approaching the levels of the sham group over time (Fig. 3*C*). The ladder-rung walking test was used to evaluate motor balance. From Day 1 post-MCAO, the MCAO group's misstep frequency was significantly higher than the sham group. However, the MCAO + HCQ treatment group showed a significant reduction in misstep frequency compared with the MCAO group by Day 3, with statistically significant differences, which persisted even at Day 21 (Fig. 3*D*).

**Figure 4.**
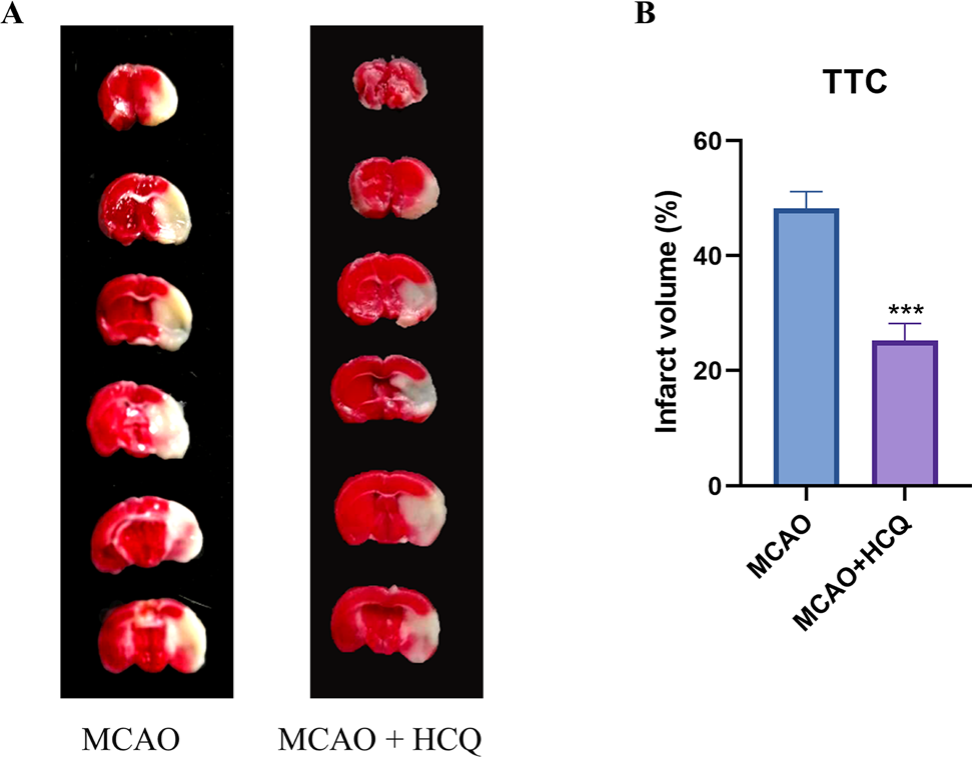
TTC staining showing the impact of HCQ on infarct volume in stroke mice. ***A***, Comparison between MCAO + HCQ and MCAO groups reveals a significant decrease in the white infarct area, which also appears darker. ***B***, ***p < 0.001 compared with the MCAO group. *n* = 8, data represented as mean ± SD.

Subsequently, mouse brain infarct volume changes were analyzed using the TTC staining technique. As depicted in Figure 4*A* and *B*, a distinct white infarct area was observable in the MCAO injury group, while the infarct volume was significantly reduced in the MCAO + HCQ treatment group. The experimental results demonstrate that HCQ can notably decrease the brain infarct volume induced by MCAO injury in mice, thereby improving the condition of brain infarction.

The aforementioned experimental findings underscore the efficacy of HCQ in alleviating motor balance and behavioral impairments in mice induced by MCAO, reducing brain infarct volume, and demonstrating a promising therapeutic effect of HCQ on ischemic stroke.

### HCQ inhibits cell pyroptosis in the cerebral cortex

**Figure 5.**
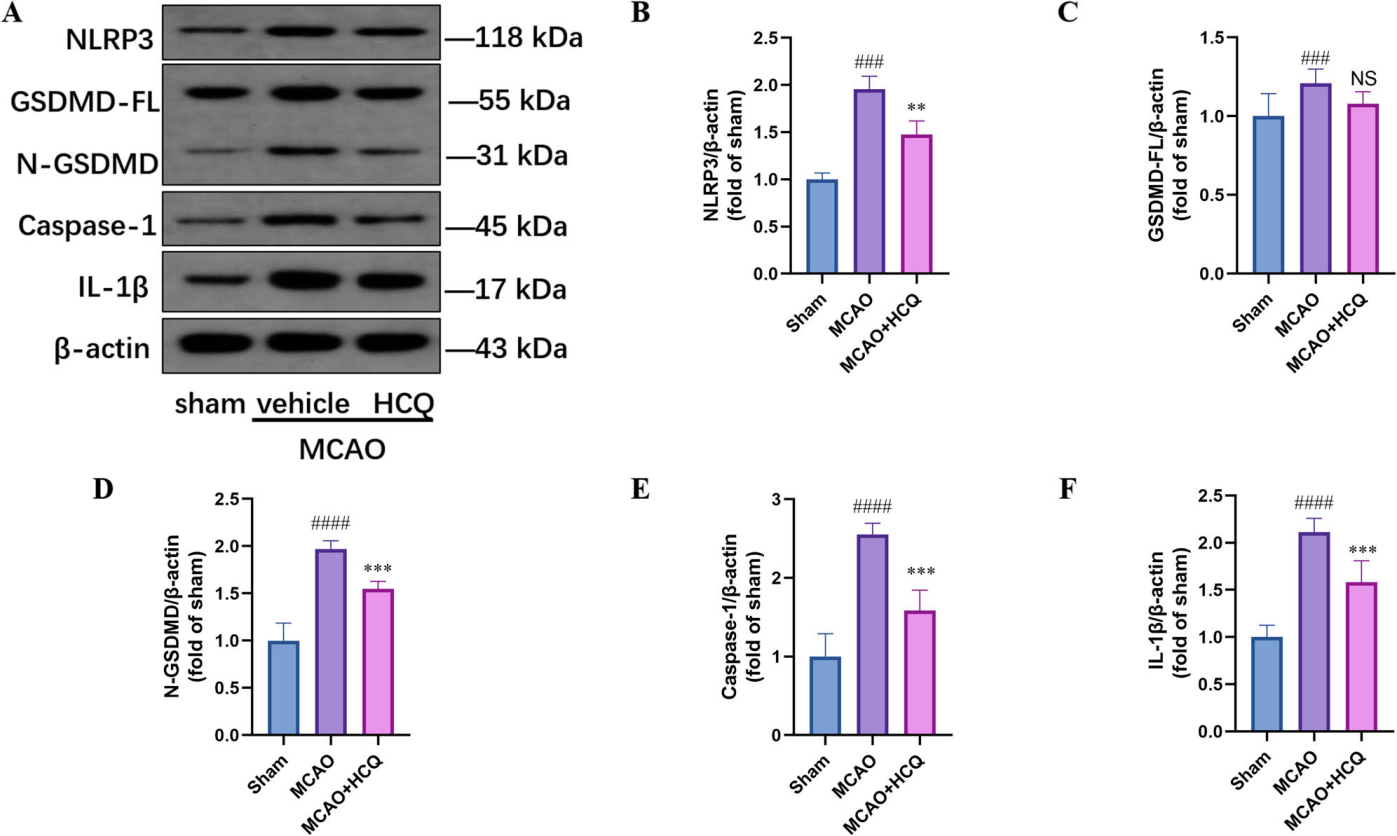
Inhibition Effect of HCQ on cell pyroptosis in mice on the third day after MCAO. ***A***, Representative images of the expression levels of NLRP3, GSDMD-FL, N-GSDMD, Caspase-1, and IL-1β in the brain tissues of mice on the third day after MCAO, detected by Western blot. β-Actin was used as the internal control. ***B–F***, Western blot quantitative analysis graphs of NLRP3, GSDMD-FL, N-GSDMD, Caspase-1, and IL-1β on the third day after MCAO. ^###^*p *< 0.001, ^####^*p *< 0.0001, compared with the Sham group; **p < 0.01, ***p < 0.001, compared with the MCAO injury group; NS indicates no statistical difference between the MCAO + HCQ and MCAO groups. *n* = 4, data presented as mean ± SD.

In the aforementioned experiment, we have confirmed that MCAO successfully induced cell pyroptosis in the mouse cerebral cortex tissue, with a peak expression of pyroptosis achieved on the third-day postischemia. Consequently, the experiment involved the examination of the cerebral cortex of mice from the sham-operated group, the MCAO injury group, and the MCAO + HCQ treatment group on the third-day postischemia using Western blot analysis. The primary proteins examined included NLRP3, GSDMD, Caspase-1, and IL-1β. As depicted in Figure 5*A–F*, the analysis results revealed a significant upregulation of the aforementioned markers in the MCAO injury group compared with the sham-operated group. Conversely, in the MCAO + HCQ treatment group, a significant downregulation of NLRP3, N-GSDMD, Caspase-1, and IL-1β proteins was observed compared with the MCAO group. These findings indicate that HCQ can inhibit cell pyroptosis in the cerebral cortex of MCAO mice.

### Major occurrence of pyroptosis in microglial cells in MCAO mouse brain tissue

**Figure 6.**
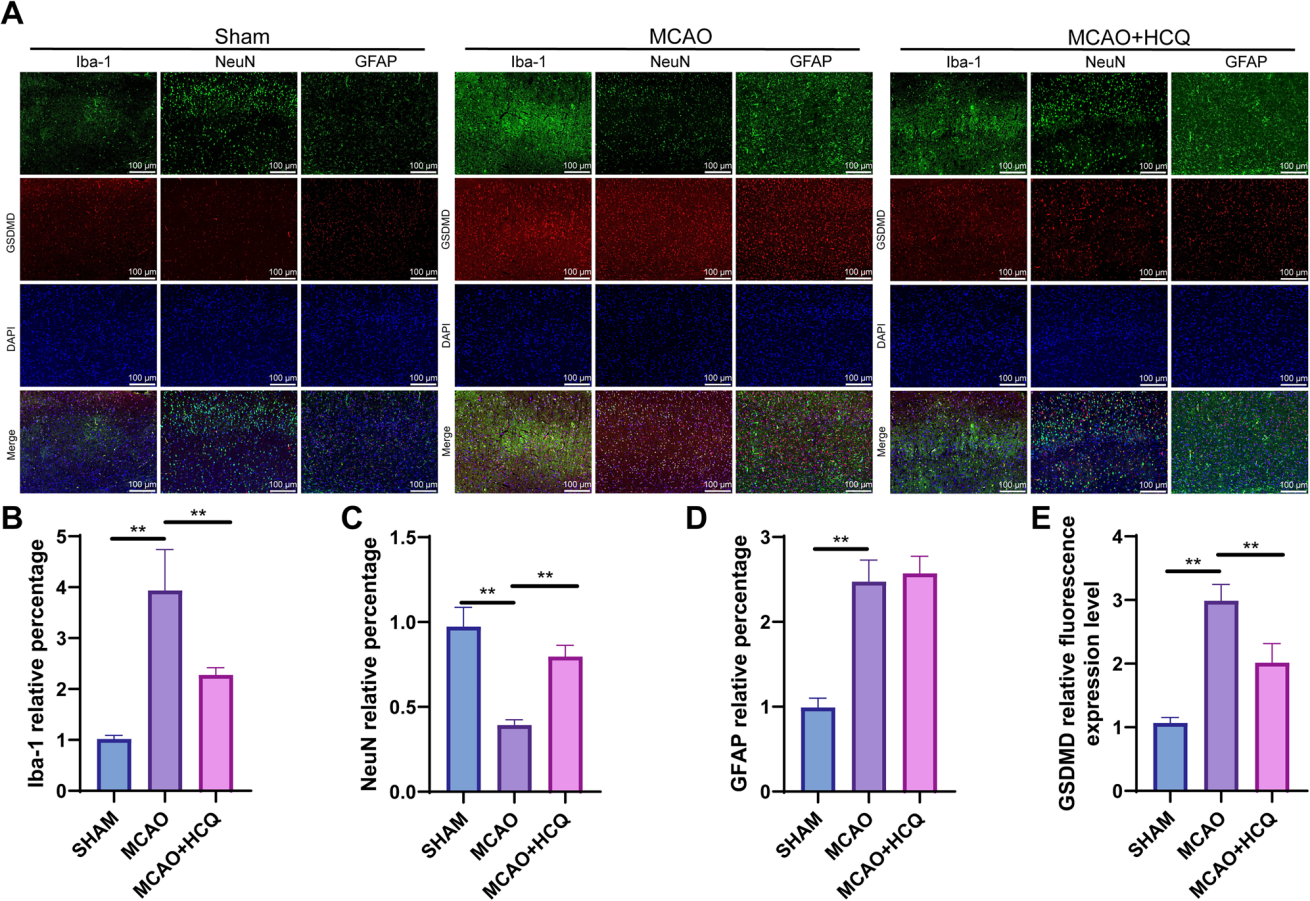
Immunofluorescence staining of cell pyroptosis in the brain ischemic area 3 d after MCAO treatment. ***A***, Colocalization imaging of pyroptosis-related protein GSDMD (in red) with Iba-1 (microglial cell marker), GFAP (astrocyte marker), and NeuN (neuronal marker). Scale bar, 100 µm. ***B–D***, Quantitative analysis of different cell types: The number of microglial cells (Iba-1) significantly increased after cerebral ischemia (***B***), while the number of neurons (NeuN) decreased (***C***), and the number of astrocytes (GFAP) increased (***D***). ***E***, Quantitative analysis of GSDMD expression.

Studies have demonstrated that brain cells primarily consist of neurons and neuroglial cells, with neuroglial cells further classified into small glial cells and astrocytes. These cell types have distinct roles in various brain regions, all of which are of paramount importance. Research indicates that GSDMD is the key initiator of pyroptosis and is the primary signaling molecule in promoting pyroptosis in microglial cells. Therefore, in order to investigate the distribution of cell apoptosis in the brain following ischemia in MCAO mice, we utilized immunofluorescence for cellular colocalization. As shown in Figure 6, compared with the sham group, the number of microglial cells (Iba-1) increased around the infarct area on the third day after cerebral ischemia in MCAO mice (Fig. 6*A*,*B*), while the number of NeuN decreased (Fig. 6*A*,*C*), and the number of GFAP increased (Fig. 6*A*,*D*), reflecting their response in repairing and protecting brain tissue after injury. However, except for the number of GFAP (Fig. 6*A*,*E*), the HCQ intervention group showed the opposite trend. Additionally, GSDMD expression was significantly higher in the cerebral cortex of MCAO mice compared with the sham group (Fig. 6*A*,*E*), whereas the level of GSDMD in the MCAO + HCQ group was markedly reduced (Fig. 6*A*,*E*). Immunofluorescence colocalization results reveal a significant expression of pyroptosis in microglia cells on the third-day postischemia, with minimal expression in neurons. Interestingly, throughout the pyroptosis process in astrocytes, there was a consistent lack of colocalization with GSDMD (Fig. 5). These findings indicate that postischemia in MCAO mice, GSDMD is predominantly expressed in small glial cells, with minimal expression in neurons.

### Inhibition effects of HCQ on the pyroptosis of microglial cells

**Figure 7.**
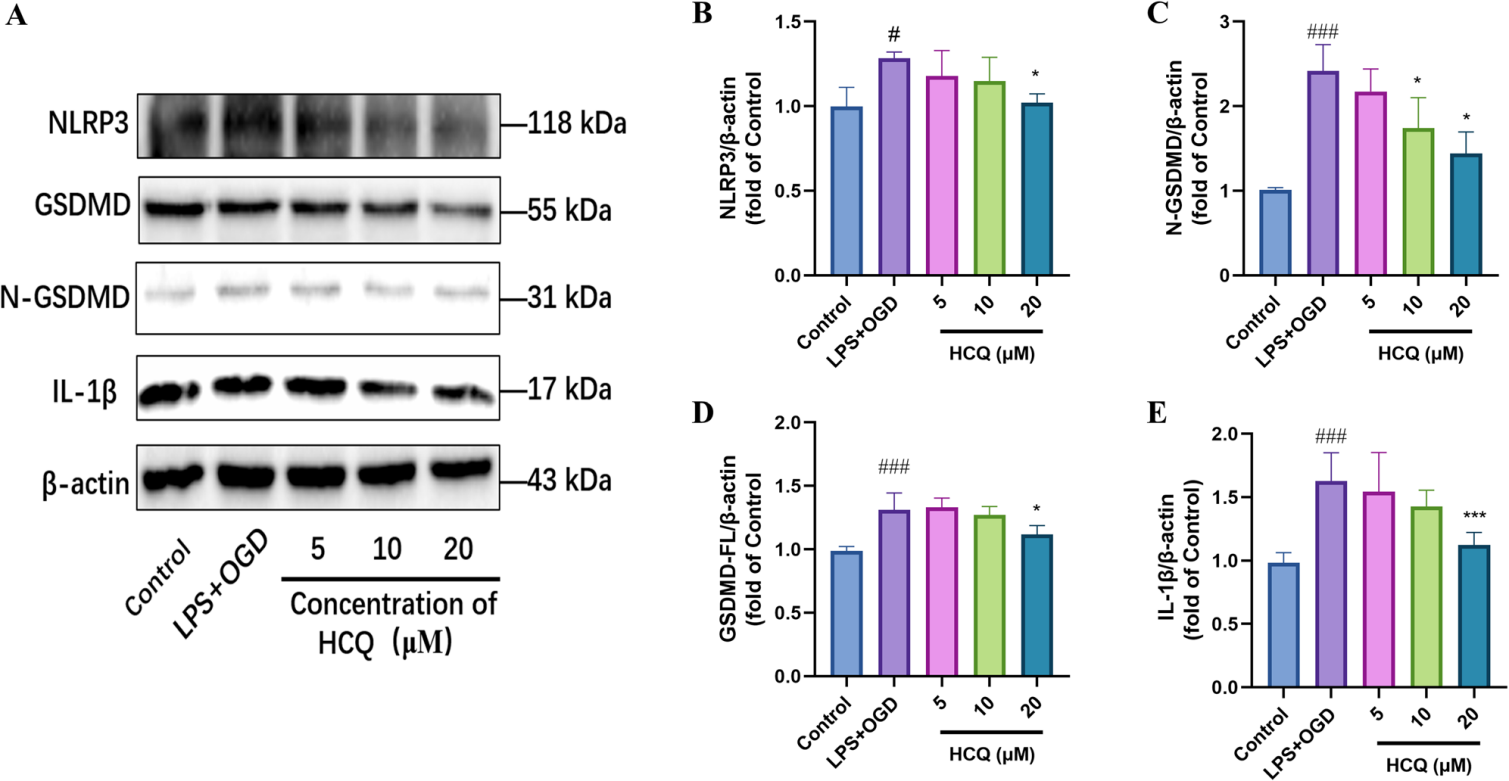
Impact of HCQ on necroptosis-related proteins in BV2 cells. ***A***, Protein immunoblot images representing the effects of different concentrations of HCQ (5, 10, 20 µM) treatment on the expression levels of NLRP3, GSDMD-FL, N-GSDMD, and the inflammatory factor IL-1β in BV2 cells detected by Western blot technique. ***B–E***, Quantitative analysis of protein immunoblots for NLRP3, GSDMD-FL, N-GSDMD, and IL-1β. ^#^*p *< 0.05, ^###^*p *< 0.001, compared with the Control group; *p < 0.05, **p < 0.01, ***p < 0.001, ns indicates no significant difference, compared with the LPS + OGD group. *n* = 4, data are presented as mean ± SD.

**Figure 8.**
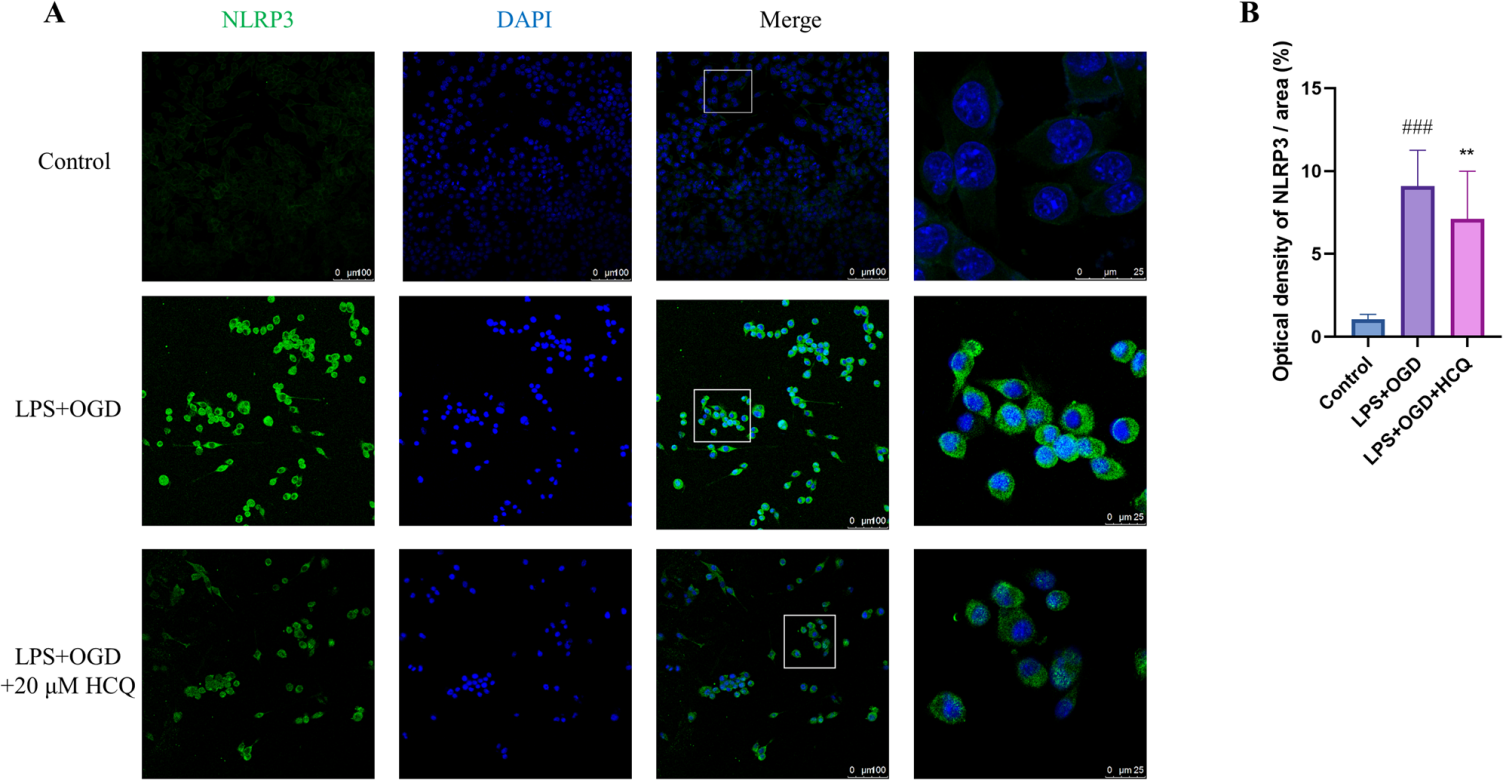
Effect of HCQ (20 µM) on the inhibition of NLRP3 inflammasome assembly in BV2 cells post LPS + OGD treatment. ***A***, Representative immunofluorescence images displaying the NLRP3 expression in BV2 cells in different groups. Scale bar, 100 µm, with a 4× magnification of the inset region showing a scale bar of 25 µm. ***B***, Proportion of NLRP3 protein-positive spots. Statistical analysis showed a significant difference ***p < 0.001 compared with the Control group; ^##^*p *< 0.01 compared with the LPS + OGD group. *n* = 4, data are presented as mean ± SEM.

In a cellular pyroptosis model (Zheng et al., 2021), the LPS + OGD group showed a significant upregulation of the inflammatory body NLRP3 and pyroptosis-related proteins GSDMD-FL, N-GSDMD, and IL-1β compared with the Control group, indicating that LPS + OGD indeed induced pyroptosis in microglial cells (Fig. 7*A–E*). Among the three groups receiving different doses, the 5 and 10 µM HCQ groups did not exhibit a noticeable downregulation effect on pyroptosis-related proteins, whereas HCQ at a concentration of 20 µM significantly decreased NLRP3 levels and suppressed the expression of GSDMD and the inflammatory factor IL-1β, as shown in Figure 7*B–E*. Finally, immunofluorescence staining was employed to detect the fluorescence expression of NLRP3 (green) in microglial cells activated after LPS + OGD treatment and treated with 20 µM HCQ. Fluorescent imaging in Figure 8 demonstrated increased NLRP3-positive spots in microglial cells following LPS + OGD treatment, significantly decreasing after 20 µM HCQ treatment. These experimental results confirm the inhibitory effect of HCQ on pyroptosis in microglial cells, indicating that a 20 µM HCQ dose effectively inhibits pyroptosis induced by LPS + OGD.

## Discussion

HCQ, a traditional antimalarial drug, has been shown to have therapeutic potential for autoimmune diseases (Ferreira et al., 2021; Grygiel-Górniak, 2022; Society for Maternal-Fetal Medicine (SMFM). Electronic address: pubs@smfm.org et al., 2023). In recent years, HCQ's neuroprotective properties have attracted growing attention from the scientific community (Koch et al., 2021; Babataheri et al., 2023). This study found that HCQ significantly inhibited the expression of pyroptosis markers GSDMD and NLRP3 in microglia, a mechanism similar to HCQ's inhibition of cell death pathways in rheumatoid arthritis. This finding expands our understanding of HCQ's role in inhibiting pyroptosis and provides new scientific evidence for its potential application in neuroprotection.

The inhibitory effect of HCQ on microglial pyroptosis was observed in an in vitro model simulating poststroke injury conditions using LPS and OGD. Previous studies have extensively investigated the role of microglia in neurological damage, such as stroke, identifying them as key cells in the progression of stroke and neurodegenerative diseases (Airas and Yong, 2022; Varma et al., 2023). Our results showed that HCQ treatment significantly reduced the expression of GSDMD and NLRP3, consistent with previous studies reporting HCQ's inhibition of similar cell death pathways in other injury models (Abdelrahman et al., 2023). This similarity highlights HCQ's potential multifunctionality in anti-cell death pathways, laying the theoretical foundation for its application in ischemic stroke treatment (Borah et al., 2021). Additionally, previous studies have demonstrated HCQ's involvement in regulating oxidative stress in cardiac tissue (Qu et al., 2020; Seydi et al., 2023). Recent research further revealed that HCQ promotes cellular repair by modulating autophagy-related pathways (Ferreira et al., 2021). These findings enrich the understanding of HCQ's diverse mechanisms and suggest that factors related to pyroptosis, such as autophagy and oxidative stress, may be involved in HCQ's role in promoting neural repair poststroke.

In this study, HCQ treatment in MCAO mice resulted in significant improvements in motor function and a reduction in infarct volume. These findings align with research demonstrating HCQ's beneficial effects on recovery following neurological injury (Zhao and Yang, 2021; Fan et al., 2023). For example, HCQ has been reported to improve neural function and mitigate cell death in spinal cord injury models (Haight et al., 2020; Brown et al., 2021). Our study further shows that HCQ directly influences neuronal death poststroke by inhibiting pyroptosis, a mechanism that has not been thoroughly explored in previous studies. This finding offers new perspectives and experimental evidence for the clinical use of HCQ in treating ischemic stroke.

Pyroptosis is crucial in ischemic stroke (Long et al., 2023; Gong et al., 2024). The regulation of this type of cell death is critical for alleviating stroke-induced pathological effects (Faria et al., 2021; Li et al., 2022; Luo et al., 2022). HCQ demonstrates potential efficacy in controlling pyroptosis by inhibiting the activation of the NLRP3 inflammasome and reducing the expression of pyroptosis markers (Yapasert et al., 2021; Luo et al., 2023). However, regulating pyroptosis in clinical and experimental settings remains challenging, as it requires precise modulation of cell death without compromising normal cell function (Büyükcavlak et al., 2022; D’Andrea et al., 2022). The experimental results suggest that HCQ's ability to inhibit pyroptosis is a rare and valuable characteristic (Tascioglu et al., 2021; Nunez et al., 2023; Zhao et al., 2024). Compared with other drugs, this feature may make HCQ a valuable tool in managing pyroptosis-related neuronal damage, particularly in facilitating cell recovery after stroke (Groot et al., 2019; Ong et al., 2021). Further research should maximize this effect and explore potential synergies with other therapeutic approaches.

The historical use of HCQ demonstrates its good tolerability and manageable side effects with long-term application, providing a basis for its use in treating ischemic stroke (Berkman and Tapson, 2021; Liu et al., 2023). Especially considering the potential need for long-term or maintenance therapy in stroke patients, the safety profile of HCQ is particularly crucial (Schwartz et al., 2021; D’Andrea et al., 2022). Additionally, the oral administration of HCQ and its widespread availability globally make it an attractive therapeutic option in low-resource settings (Raj et al., 2021; Singh et al., 2022). However, despite the relatively fewer side effects of HCQ, caution must be exercised regarding its potential long-term risks, such as retinal toxicity, necessitating regular monitoring and evaluation in clinical practice (Dima et al., 2022; Bykowski et al., 2023; Rao et al., 2023). Furthermore, studying the effects and safety of HCQ in patient populations of different races and genetic backgrounds is an important aspect of future research (Ismaila et al., 2021; Narayanasamy et al., 2022; Klebanov et al., 2023). Through such research, we can enhance our understanding of the mechanisms of action of HCQ and optimize its utilization strategies for more precise and effective clinical applications.

Regarding the application of HCQ in other neurological disorders, studies have shown its potential benefits in treating multiple sclerosis and Parkinson's disease (Koch et al., 2021; Airas and Yong, 2022; Zhang et al., 2023). Similar to ischemic stroke, these diseases involve neuronal damage (Berkman and Tapson, 2021; de Carvalho and Shoenfeld, 2021). The use of HCQ in these cases suggests its broad neuroprotective potential, providing additional scientific support for its application in treating ischemic stroke (Ferreira et al., 2021; Stack and McCarthy, 2021; Trindade et al., 2021). Moreover, some recent studies have explored the potential impact of HCQ on neurogenesis, a key factor in the stroke recovery process. These findings demonstrate the multifaceted role of HCQ as a versatile drug in treating neurological disorders, thus offering broader scientific and clinical implications to this study. Future research can further investigate the specific mechanisms of action of HCQ in these areas, particularly how it influences neuro repair and regeneration processes, providing important insights for comprehensive therapeutic strategies for stroke and other neurological disorders.

This study systematically evaluated the impact of HCQ on microglial cell pyroptosis postischemic stroke in vitro and in vivo models, confirming that HCQ significantly improves neurobehavioral performance and reduces infarct volume. This conclusion not only deepens our understanding of the pathophysiology of ischemic stroke but also provides scientific evidence for HCQ as a potential therapeutic agent for such conditions. Compared with treatments like tPA, HCQ offers the advantage of low drug cost, providing a practical basis for its clinical application. However, this study has some limitations, such as the choice of model, the use of a single mouse strain, and the experimental conditions, which may affect the generalizability of the results. Future research should further validate HCQ's effects in more diverse animal models and human cells, exploring its optimal dosage, administration methods, and potential clinical benefits when combined with other drugs. We are also aware of the potential risk of retinal toxicity associated with long-term HCQ use (Martín-Iglesias et al., 2021; Yusuf et al., 2023). Future studies should focus on the potential retinal toxicity of prolonged HCQ treatment in stroke patients. Additionally, deeper investigations into HCQ's mechanisms of action, as well as the development of new combination therapies to mitigate retinal toxicity, will help optimize the use of this drug in treating ischemic stroke and other neurological disorders.

### Conclusion

**Figure 9.**
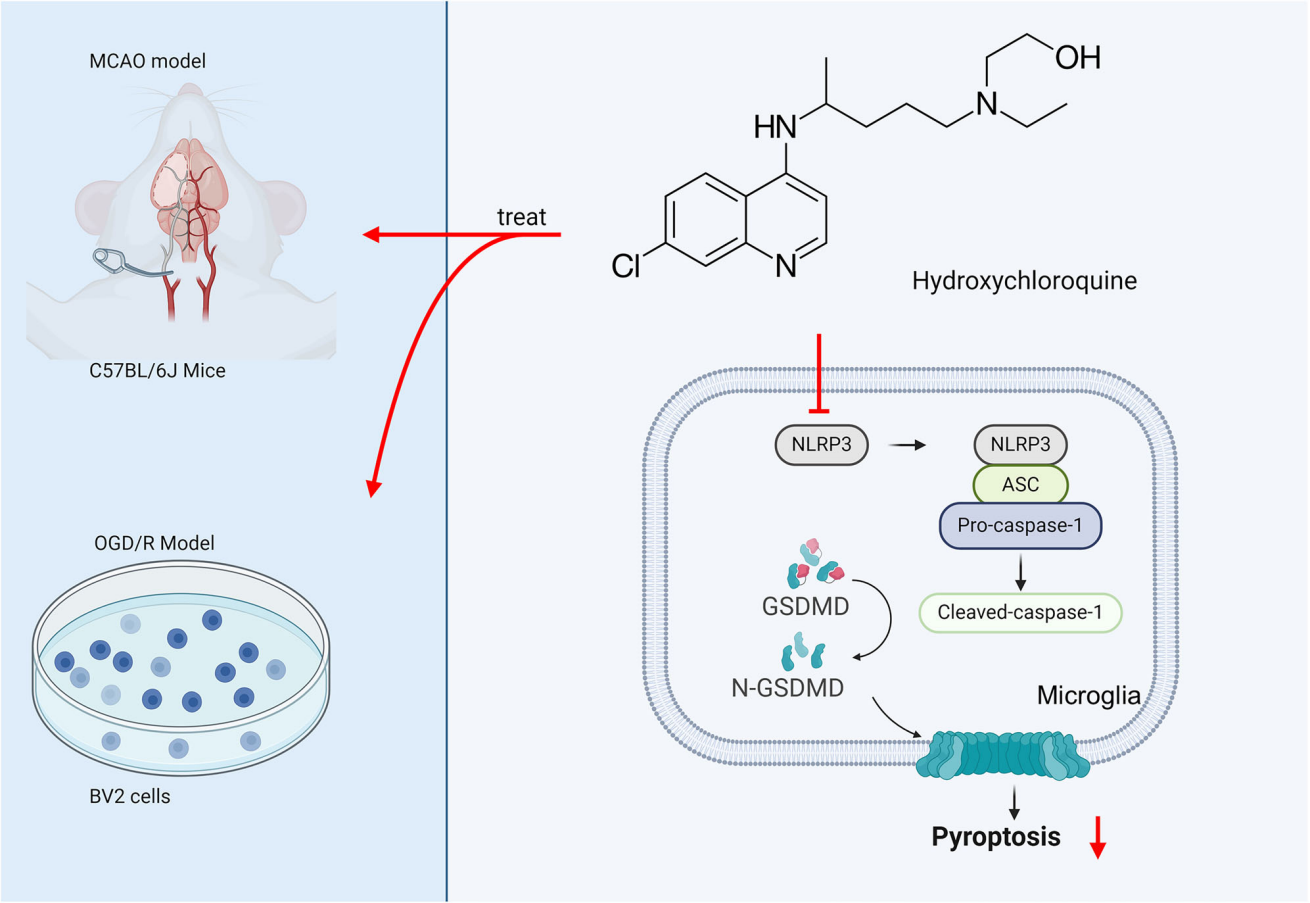
Potential neuroprotective effects of HCQ in ischemic stroke: inhibiting cell apoptosis and improving neural function.

The comprehensive analysis of the aforementioned research findings reveals that HCQ, as a potential therapeutic agent, exhibits significant neuroprotective effects in ischemic stroke. By effectively inhibiting cell pyroptosis, HCQ can ameliorate neuronal functional damage and reduce cerebral infarction volume, thus holding crucial potential in disease treatment (Fig. 9). These findings provide strong scientific evidence for further research and application of HCQ as a therapeutic agent for ischemic stroke. Consequently, the prospects of HCQ in neuroprotection are promising, warranting further exploration in clinical practice.

## Data Availability

The original contributions presented in the study are included in the article/supplementary materials, further inquiries can be directed to the corresponding authors.

## Synthesis

Reviewing Editor: Mikko Airavaara, Helsingin yliopisto - Viikin kampus Decisions are customarily a result of the Reviewing Editor and the peer reviewers coming together and discussing their recommendations until a consensus is reached. When revisions are invited, a fact-based synthesis statement explaining their decision and outlining what is needed to prepare a revision will be listed below. The following reviewer(s) agreed to reveal their identity: NONE.

The paper " Inhibition of Pyroptosis by Hydroxychloroquine as a Neuroprotective Strategy in Ischemic Stroke " presents a study investigating the therapeutic role of hydroxychloroquine (HCQ) on ischemic stroke pathology. The study employs a thorough experimental design that includes both in vivo (mouse model) and in vitro (cell culture) components. The use of multiple behavioral tests (mNSS, rotarod, grip strength, and foot fault test) to assess neurobehavioral function is commendable as it provides a well-rounded evaluation of motor function impairments post-stroke. The authors claim that HCQ inhibits pyroptosis, thereby improving motor function and reducing infarct volume in the MACO mouse model. The article presents clear evidence of claims made by the authors and systematically presents the findings.

Major comments:

1. Though the study focuses on HCQ effect on pyroptosis- The study does not compare HCQ with other neuroprotective agents or standard stroke treatments (e.g., tPA). A comparative analysis would help position HCQ's effectiveness relative to existing therapies, providing a clearer understanding of its potential role in clinical practice.

2. While the authors investigate the inhibition of pyroptosis by HCQ, however, they do not discuss deeply the molecular signaling pathways downstream of NLRP3 and GSDMD that might be affected by HCQ. It is suggest adding downstream signaling pathways or intermediates that might be involved in HCQ's action, such as autophagy, apoptosis, or oxidative stress pathways, to the discussion.

3. While the study highlights HCQ's neuroprotective effects, it does not sufficiently address the long-term safety concerns associated with HCQ use, such as retinal toxicity. The discussion could benefit from a more detailed consideration of these potential risks, especially since stroke patients may require long-term treatment.

4. The study relies on a single mouse model (C57BL/6), which may not represent the diverse biological responses in other strains or species. How do the findings differ from other models? Comparative studies are crucial to determine whether HCQ offers any distinct advantages or whether it could be used in combination with existing therapies, for example, thrombolytics and anticoagulants for enhanced outcomes.

5. The article frequently cites studies on the anti-inflammatory effects of HCQ in diseases other than stroke, which, while relevant, might be overemphasized compared to direct evidence in stroke models. The article could benefit from a more focused review of studies specifically investigating HCQ in neurological contexts, particularly in relation to stroke and pyroptosis.

6. Figure 3 after the stroke significance in MCAO + HCQ groups are not systematically significant. Is this associated with inter animal variations? It would be nice to depict either boxplot or violin plot to see the distribution.

7. Figure 4b significance can be shown by stars instead of # to be consistent with other figures

8. In Fig2, authors predicted HCQ target proteins associated with ischemic stroke with bioinformatic method. Which model or bioinformatic method did authors use for prediction? Which data set did authors use for prediction (mice versus human)? Authors should include all important details in the paper.

9. For HCQ treatment post stroke, when did the authors inject HCQ? Does HCQ show different protective effects at 1-day, 3-day or 5-day post stroke?

10, In Fig6, which brain region was included? Authors should also include "Sham" group and "MCAO+HCQ" group and compare GSDMD expression in different conditions.

11., Scale bar in all images should be labeled clearer.

## Author Response

Synthesis of Reviews:

Computational Neuroscience Model Code Accessibility Comments for Author (Required):

N/A Synthesis Statement for Author (Required):

The paper " Inhibition of Pyroptosis by Hydroxychloroquine as a Neuroprotective Strategy in Ischemic Stroke " presents a study investigating the therapeutic role of hydroxychloroquine (HCQ) on ischemic stroke pathology. The study employs a thorough experimental design that includes both in vivo (mouse model) and in vitro (cell culture) components. The use of multiple behavioral tests (mNSS, rotarod, grip strength, and foot fault test) to assess neurobehavioral function is commendable as it provides a well-rounded evaluation of motor function impairments post-stroke. The authors claim that HCQ inhibits pyroptosis, thereby improving motor function and reducing infarct volume in the MACO mouse model. The article presents clear evidence of claims made by the authors and systematically presents the findings.

Response:

Thank you for your thoughtful review of our manuscript, We sincerely appreciate your positive feedback regarding the design of our study, including both in vivo and in vitro components, as well as your kind recognition of the behavioral tests we employed to assess motor function impairments. Your comments affirm our efforts to systematically demonstrate the neuroprotective effects of hydroxychloroquine (HCQ) through the inhibition of pyroptosis in the MACO mouse model. Based on your feedback, we have further refined and supplemented the manuscript to ensure that the presentation of the research findings is more rigorous and comprehensive.

Major comments:

1. Though the study focuses on HCQ effect on pyroptosis- The study does not compare HCQ with other neuroprotective agents or standard stroke treatments (e.g., tPA). A comparative analysis would help position HCQ's effectiveness relative to existing therapies, providing a clearer understanding of its potential role in clinical practice.

Response:

We acknowledge the importance of comparing hydroxychloroquine (HCQ) with other neuroprotective agents or standard treatments such as tPA. However, since this study primarily focuses on the mechanism of HCQ in inhibiting pyroptosis, a detailed comparison with tPA or other drugs falls beyond the scope of this research. We have now included a comparison between HCQ and other therapies, such as tPA, in the discussion section as follows: " Compared to treatments like tPA, HCQ offers the advantage of low drug cost, providing a practical basis for its clinical application. " 2. While the authors investigate the inhibition of pyroptosis by HCQ, however, they do not discuss deeply the molecular signaling pathways downstream of NLRP3 and GSDMD that might be affected by HCQ. It is suggest adding downstream signaling pathways or intermediates that might be involved in HCQ's action, such as autophagy, apoptosis, or oxidative stress pathways, to the discussion.

Response:

We have expanded the discussion to include more details on molecular pathways downstream of NLRP3 and GSDMD that may be affected by HCQ. Specifically, we have discussed the potential involvement of autophagy, apoptosis, and oxidative stress pathways, as suggested.

3. While the study highlights HCQ's neuroprotective effects, it does not sufficiently address the long-term safety concerns associated with HCQ use, such as retinal toxicity. The discussion could benefit from a more detailed consideration of these potential risks, especially since stroke patients may require long-term treatment.

Response:

We have addressed the long-term safety concerns associated with HCQ, particularly retinal toxicity, in the discussion. This point is now emphasized in relation to the clinical application of HCQ for stroke patients, and we suggest regular monitoring as a precautionary measure.

4. The study relies on a single mouse model (C57BL/6), which may not represent the diverse biological responses in other strains or species. How do the findings differ from other models? Comparative studies are crucial to determine whether HCQ offers any distinct advantages or whether it could be used in combination with existing therapies, for example, thrombolytics and anticoagulants for enhanced outcomes.

Response:

We have acknowledged the limitation of using only a single mouse strain (C57BL/6) and added a statement in the limitations section of the manuscript emphasizing the need for future studies to include other strains or species to ensure broader applicability. We also agree that comparative studies are essential to evaluate the potential of combining HCQ with existing therapies, such as thrombolytic agents.

5. The article frequently cites studies on the anti-inflammatory effects of HCQ in diseases other than stroke, which, while relevant, might be overemphasized compared to direct evidence in stroke models. The article could benefit from a more focused review of studies specifically investigating HCQ in neurological contexts, particularly in relation to stroke and pyroptosis.

Response:

We have revised the manuscript to enhance the discussion on HCQ in the context of stroke, focusing specifically on its role in the neurological field, particularly in ischemic stroke and pyroptosis. At the same time, we have reduced the emphasis on HCQ's anti-inflammatory effects in other diseases.

6. Figure 3 after the stroke significance in MCAO + HCQ groups are not systematically significant. Is this associated with inter animal variations? It would be nice to depict either boxplot or violin plot to see the distribution.

Response:

We have updated Figure 3 with boxplots to depict the distribution more clearly and address any potential inter-animal variations. We believe this change provides a better visualization of the data.

7. Figure 4b significance can be shown by stars instead of # to be consistent with other figures Response:

We have modified the significance markers in Figure 4B to stars (*) instead of "#" to ensure consistency across the figures.

8. In Fig2, authors predicted HCQ target proteins associated with ischemic stroke with bioinformatic method. Which model or bioinformatic method did authors use for prediction? Which data set did authors use for prediction (mice versus human)? Authors should include all important details in the paper.

Response:

We have provided more detailed information about the bioinformatics methods in the "Identification of HCQ Potential Target Proteins" section. Specifically, we utilized SwissTargetPrediction, DrugBank, and PharmMapper, and we clarified that the datasets used for predictions are based on human protein interactions.

9. For HCQ treatment post stroke, when did the authors inject HCQ? Does HCQ show different protective effects at 1-day, 3-day or 5-day post stroke? Response:

We have now supplemented additional information regarding the timing of HCQ injection after stroke. Specifically, we stated in the Methods section under "Animal Grouping for Experiment": "The treatment group received an intraperitoneal injection of 50 mg/kg HCQ immediately following MCAO surgery, administered once daily. No significant difference in protective effects was observed between treatments administered on days 1, 3, and 5 post-stroke." 10, In Fig6, which brain region was included? Authors should also include "Sham" group and "MCAO+HCQ" group and compare GSDMD expression in different conditions.

Response:

We have added more details regarding the brain regions studied. Specifically, "Brain tissue was extracted from the cerebral cortex." Additionally, we have updated Figure 6 to include the "Sham" and "MCAO + HCQ" groups to compare GSDMD expression under different conditions.

11., Scale bar in all images should be labeled clearer.

Response:

We have ensured that all scale bars in the images are now labeled more clearly to improve clarity and accuracy.
